# Leukemia-associated truncation of granulocyte colony-stimulating factor receptor impacts granulopoiesis throughout the life-course

**DOI:** 10.3389/fimmu.2022.1095453

**Published:** 2023-01-10

**Authors:** Vilasha Bulleeraz, Michelle Goy, Faiza Basheer, Clifford Liongue, Alister C. Ward

**Affiliations:** ^1^ School of Medicine, Deakin University, Geelong, VIC, Australia; ^2^ Institute for Mental and Physical Health and Clinical Translation (IMPACT), Deakin University, Geelong, VIC, Australia

**Keywords:** cytokine receptors, G-CSFR, leukemia, neutropenia, zebrafish

## Abstract

**Introduction:**

The granulocyte colony-stimulating factor receptor (G-CSFR), encoded by the *CSF3R* gene, is involved in the production and function of neutrophilic granulocytes. Somatic mutations in *CSF3R* leading to truncated G-CSFR forms are observed in acute myeloid leukemia (AML), particularly those subsequent to severe chronic neutropenia (SCN), as well as in a subset of patients with other leukemias.

**Methods:**

This investigation introduced equivalent mutations into the zebrafish *csf3r* gene *via* genome editing and used a range of molecular and cellular techniques to understand the impact of these mutations on immune cells across the lifespan.

**Results:**

Zebrafish harboring truncated G-CSFRs showed significantly enhanced neutrophil production throughout successive waves of embryonic hematopoiesis and a neutrophil maturation defect in adults, with the mutations acting in a partially dominant manner.

**Discussion:**

This study has elucidated new insights into the impact of G-CSFR truncations throughout the life-course and created a *bone fide* zebrafish model for further investigation.

## 1 Introduction

The granulocyte colony-stimulating factor receptor (G-CSFR) is a key regulator of neutrophil production, or granulopoiesis, by impacting on the proliferation and differentiation of precursors as well as enhancing neutrophil survival (1, 2). The G-CSFR is activated by the cytokine G-CSF, which is produced developmentally (3), but also in response to injury and infection to stimulate so-called ‘emergency’ granulopoiesis (2). This cytokine is also used clinically in situations where neutrophil numbers are low (4).

Acquired somatic mutations in *CSF3R*, the gene encoding G-CSFR, have been found in a variety of leukemias (5). These include nonsense and frameshift mutations within exon 17 that serve to truncate the G-CSFR intracellular domain that are observed in acute myeloid leukemia (AML), particularly subsequent to severe chronic neutropenia (SCN) (6, 7), but also *de novo* and relapsed forms of the disease (8–10). Similar mutations have also been identified in chronic neutrophilic leukemia (CNL), atypical chronic myelogenous leukemia (aCML) and chronic myelomonocytic leukemia (CMML) patients (9, 11–14), although these are usually co-incident with alternative constitutively-activating G-CSFR mutations (15). The locations of the receptor truncations are quite variable, being found from position 738 through to 819 (5, 7–14). They lead to hyperresponsiveness to G-CSF, enhanced signaling particular of STAT5, and increased proliferation (16–18), with the ability to mediate leukemic transformation *in vitro* (10). Various mouse models of G-CSFR truncation mutations have been generated by different gene targeting approaches (19–21). While all exhibited enhanced responsiveness to G-CSF and altered receptor internalization, they were variable with respect to their effects on steady-state neutrophil levels.

Zebrafish has proven to be a robust experimental platform for the study of blood and immune cell development and its perturbation in disease (22). Zebrafish granulopoiesis occurs in multiple waves like mammals (23). This includes a primitive wave generating myeloid precursors in the rostral blood island that differentiate into neutrophils as they migrate across the yolk sac and a definitive wave that ultimately produces neutrophils from the kidney marrow, the equivalent of mammalian bone marrow (24). Zebrafish has been shown to have a structurally and functionally conserved *csf3r* gene (3), which contributes to neutrophil production throughout the life-course (25, 26). The zebrafish *csf3r* gene was targeted with CRISPR-Cas9 to generate truncating hyperresponsive G-CSFR mutations based on those observed in leukemia. These *csf3r* mutant fish possessed enhanced numbers of neutrophils during primitive and definitive hematopoiesis, but in adulthood neutrophil numbers were normal but maturation was reduced. Together these data identify on-going impacts of G-CSFR truncations on neutrophil production, and describe a useful *in vivo* model for further studies.

## 2 Materials and methods

### 2.1 Fish husbandry

Wild-type and Tg(*mpo::GFP*) zebrafish, in which neutrophils are fluorescently marked (27), were maintained using standard husbandry practices (28) in a Techniplast aquarium system at 28.5°C with pH 7.0, ammonia <1.5 ppm, nitrite <3 ppm, nitrate <30 ppm and conductivity at 500 µS on a 14 h/10 h light/dark cycle and fed twice daily. Embryos were manually spawned and maintained at 28.5°C in petri dishes containing aquarium water and 0.00005% (w/v) methylene blue. At 8 h post-fertilization (hpf) this was replaced with aquarium water containing 0.003% (w/v) 1-phenyl-2-thio-urea (PTU) to inhibit pigmentation and maintain embryo transparency.

### 2.2 Genetic manipulation and analysis

Wild-type embryos at the 1 cell stage were injected with 12.5 ng/μL CRISPR guide RNA (gRNA) that targeted a sequence within *csf3r* exon 16 (CTGTTAGCAGGAGACGAGCC) in order to recapitulate human truncation mutations and 100 pg/nL Cas9 in sterile nuclease-free water along with 1:16 vol:vol 1% (w/v) phenol red and raised to adulthood. These were out-crossed with wild-type fish and carriers of relevant mutant alleles identified from fin clips obtained under anesthesia with benzocaine. Genomic DNA was isolated with QuickExtract following the manufacturer’s instructions, and subjected to PCR with *csf3r*-specific primers for High Resolution Melt (HRM) analysis (29) with primers 5’-ATTCCTCCAACCTCCAGC and 5’-CAGAGAAGCGGTTCAGTGC using Precision Melt Supermix and Analysis Software (BioRad) to identify potential mutants that were confirmed by Sanger sequencing using the primers 5’-CAGTGCTGGTGTATCTGTCCC and 5’-GCGAGTTAGATGTGATTGACC at the Australian Genome Research Facility. These founder fish were further out-crossed before being in-crossed to generate homozygous mutant fish. One allele was also crossed onto the Tg(*mpo::GFP*) (27) background. In some experiments *in vitro* transcribed *csf3a* mRNA was injected into 1-8 cell stage embryos to stimulate the G-CSFR as described (30), since the encoded ligand appears more potent than that produced by the alternate orthologue *csf3b* (31).

### 2.3 Whole-mount *in situ* hybridization

Embryos were collected at appropriate time points reflecting primitive (22 hpf) and definitive (5 dpf) waves of hematopoiesis (24) and anesthetized with 0.4 mg/mL benzocaine before fixation with 4% (w/v) paraformaldehyde in phosphate-buffered saline. Fixed embryos were stored at 4°C for at least 1 day, after which embryos were dehydrated with 100% (v/v) methanol for long-term storage at -20°C. Rehydrated embryos were subjected to WISH with anti-sense DIG-labeled probes specific for blood and immune cell lineages as described (32, 33). Stained embryos were mounted in 2% w/v methylcellulose and visualized using MVX10 monozoom microscope with a 1 × MVXPlan Apochromat lens (NA = 0.25) with an Olympus DP74 camera. Quantitation was achieved by enumeration of individual cells stained with the probe or measuring the area of staining using ImageJ software in a blind fashion on images taken on a dissecting microscope. Data were analyzed for significance with a Student’s *t*-test, using Welch’s correction where necessary.

### 2.4 Reverse-transcription polymerase chain reaction

Total RNA was extracted from 20-30 pooled zebrafish embryos with RNeasy Mini Kit (Qiagen) following the manufacturer’s protocol, and subjected to quantitative real-time reverse-transcriptase PCR (qRT^2^-PCR) with the primers for *actb* (5’-TGGCATCACACCTTCTAC and 5’- AGACCATCACCAGAGTCC), and *csf3r* (5’- CAGAGAAGCGGTTCAGTGC and 5’-ATTCCTCCAATCCTCCAGC). Data was normalized relative to *actb* and fold-change in *csf3r* expression calculated using the ΔΔCT method (34).

### 2.5 *Ex vivo* analyses

Adult zebrafish were euthanized with benzocaine and blood and kidney collected. Kidneys from fish on the Tg(*mpo::GFP*) background were placed in ice-cold phosphate-buffered saline supplemented with 1 mM EDTA and 2% (v/v) fetal calf serum and passage through a 40 μm sieve. These isolated kidney cells were analyzed using a BD FACSCantoII analyzer with lineages identified in a SSC/FSC plot, and GFP^+^ neutrophils identified in the FITC channel, while apoptosis was assessed using a PE-Annexin V/7-AAD apoptosis detection kit (Biolegend). A minimum of 100,000 events were collected for each sample using FACSCanto II flow cytometer (BD Biosciences) and analyzed using BD FACSDiva software (v6.0). Neutrophils were sorted on a FACSAriaIII (BD Biosciences) for further analysis. Cytospin preparations of blood and sorted GFP+ neutrophils were stained with Giemsa (Sigma) and slides viewed on a Leica DME stereomicroscope and imaged with a DP70 camera and differential counts performed, with overall cell and nuclear morphology used to stage neutrophils. Data were analyzed for significance with a Student’s *t*-test.

## 3 Results

### 3.1 Generation of zebrafish *csf3r* mutants based on human hyperresponsive truncation mutations

**Figure 1:**
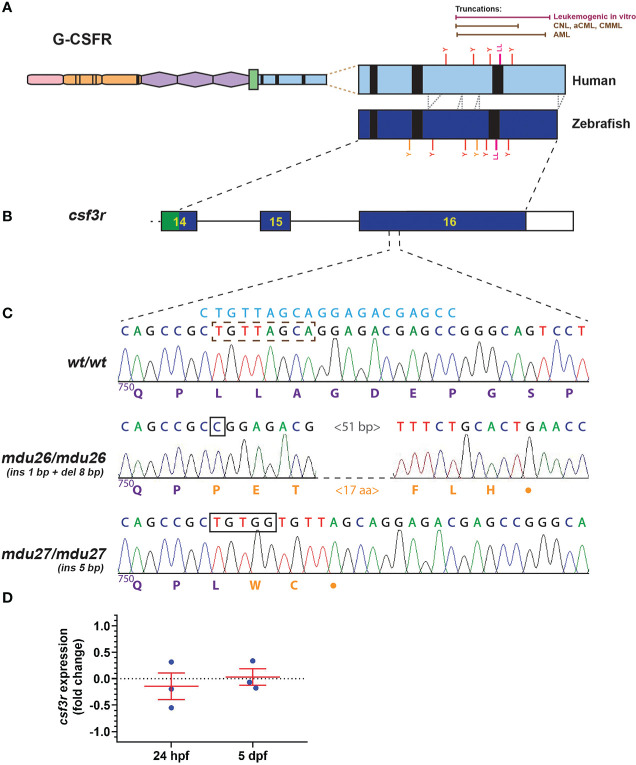
Generation of leukemia-derived G-CSFR truncation mutants. Schematic diagram of the G-CSFR showing the extracellular immunoglobulin domain (pink), cytokine receptor homology domain (orange) and fibronectin type III-like domains (purple), transmembrane region (green), and intracellular region (blue) containing Box 1-3 (black rectangles). The intracellular region is expanded to show tyrosine (Y) residues and a di-leucine motif (LL) in both human and zebrafish proteins, with the relative positions of truncation mutations found in various human leukemias or demonstrated to be leukemogenic *in vitro* shown above **(A)**. Exons 14-16 of the zebrafish *csf3r* gene encoding the intracellular region presented as numbered boxes with connecting lines depicting introns **(B)**. Sequence traces of the indicated part of *csf3r* showing homozygous wild-type (*wt/wt*) and mutant zebrafish, with nucleotide sequence above and encoded protein sequences below, in purple for native and orange for *de novo* sequence **(C)**. The gRNA target site is shown above in blue, with deleted nucleotide sequences indicated with a brown dotted box and inserted sequences shown with black boxes. The *csf3r^mdu26^* (*mdu26*) allele represents a complex 1 bp insertion/8 bp deletion and the *csf3r^mdu27^* (*mdu27*) allele a 5 bp insertion both of which cause a frameshift resulting in translation from an alternative reading frame followed by a stop codon. Gene expression analysis of *csf3r* in pooled WT and mutant embryos at the indicated timepoints presented as fold-change (log_2_) relative to *actb* **(D)**, showing mean and SEM (not significant, n=3).

Truncation mutations affecting the human G-CSFR intracellular domain have been observed in AML, commencing from position 739 through 819 (5, 8–10), and in other leukemias from 738 through 791 (9, 11–14), with truncations across this range able to mediate leukemic transformation *in vitro* (10) (**Figure 1A**). The intracellular domain is largely conserved in the zebrafish G-CSFR (**Figure 1A**), and so exon 16 of the zebrafish *csf3r* gene was targeted by genome editing using CRISPR/Cas9 (35) to recapitulate the human mutations (**Figure 1B**). A guide RNA (gRNA) was designed targeting sequences encoding a di-leucine motif shown to play an important role in G-CSFR internalization (17) (**Figure 1C**) that lies roughly in the middle of the AML mutants (**Figure 1C**). Wild-type embryos injected with this gRNA and Cas9-encoding mRNA at the one cell stage were raised to adulthood, with their progeny subjected to HRM and Sanger sequencing to identify potential *csf3r* mutations. A total of four *csf3r* mutant alleles were identified with fish carrying these alleles out-crossed before the resultant carrier progeny were in-crossed with the mutations clearly evident upon sequencing of homozygote carriers of each allele. Two alleles were chosen: *mdu26* containing a complex indel (1 bp insertion and 8 bp deletion) and *mdu27* containing a 5 bp insertion (**Figure 1C**). Both mutations caused a frameshift followed by a stop codon rendering the intracellular domain truncated, specifically P751fs23* for *mdu26* and L752fs2* for *mdu27*, including the complete or partial loss of the di-leucine motif in each case. Of the other alleles, one had a 1 bp deletion leading to P751fs25* and so almost identical to *mdu26*, while the other had a 17 bp insertion and 2 bp deletion resulting in a 5 amino acid insertion but no truncation, and so *mdu26* and *mdu27* were chosen for further study. The *mdu27* allele was crossed with Tg(*mpo:GFP*) fish in which neutrophils are fluorescently marked (27). Expression of *csf3r* was found to be similar in wild-type and mutant embryos at several timepoints (**Figure 1D**), ruling out any significant nonsense-mediated decay (36).

### 3.2 Impact of G-CSFR truncation mutation on embryonic primitive hematopoiesis

**Figure 2:**
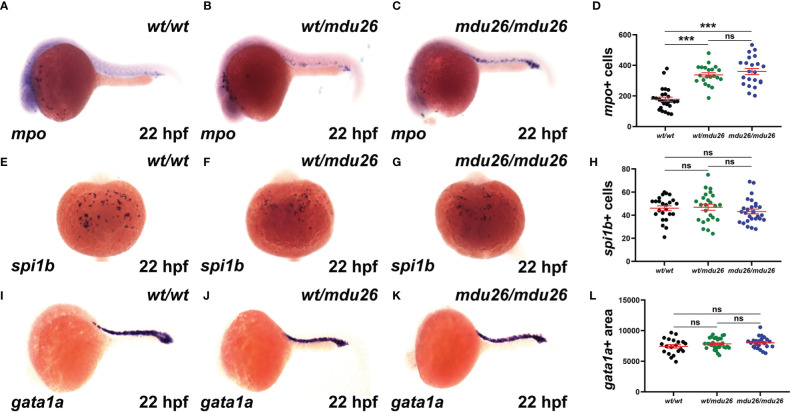
Effect of G-CSFR truncation mutation on primitive hematopoiesis. Wild-type (*wt/wt*), heterozygous (*wt/mdu26*) and homozygous (*mdu26/mdu26*) mutant *csf3r* embryos were subjected to WISH at 22 hpf with *mpo* **(A–C)**, *spi1b* **(E–G)** and *gata1a* **(I–K)**, with representative images shown. Individual embryos were assessed for the number of *mpo* ^+^ **(D)** or *spi1b* ^+^ **(H)** cells or the area of *gata1a* **(L)** staining, with mean and SEM in red and level of statistical significance indicated (***: *p* < 0.001, ns: not significant; n=20-30).

Primitive hematopoiesis commences around 12 hpf in zebrafish and is well established by 22 hpf (37). Both heterozygous *csf3r^wt/mdu26^* and homozygous *csf3r^mdu26/mdu26^* embryos showed increased numbers of cells expressing *mpo*, a marker of mature neutrophils (38), compared to *csf3r^wt/wt^* embryos (**Figures 2A–D**). A similar increase in *mpo* was observed in embryos homozygous for the other allele (*csf3r^mdu27/mdu27^*) compared to *csf3r^wt/wt^* embryos (**Supplementary Figures 1A–C**). In contrast, no differences were seen in the number of cells expressing *spi1b*, a marker of myeloid precursors (39) (**Figures 2E–H**) or the area of expression for *gata1a*, a marker of erythrocyte precursors (40) (**Figures 2I–L**).

### 3.3 Impact of G-CSFR truncation mutation on embryonic definitive hematopoiesis

**Figure 3:**
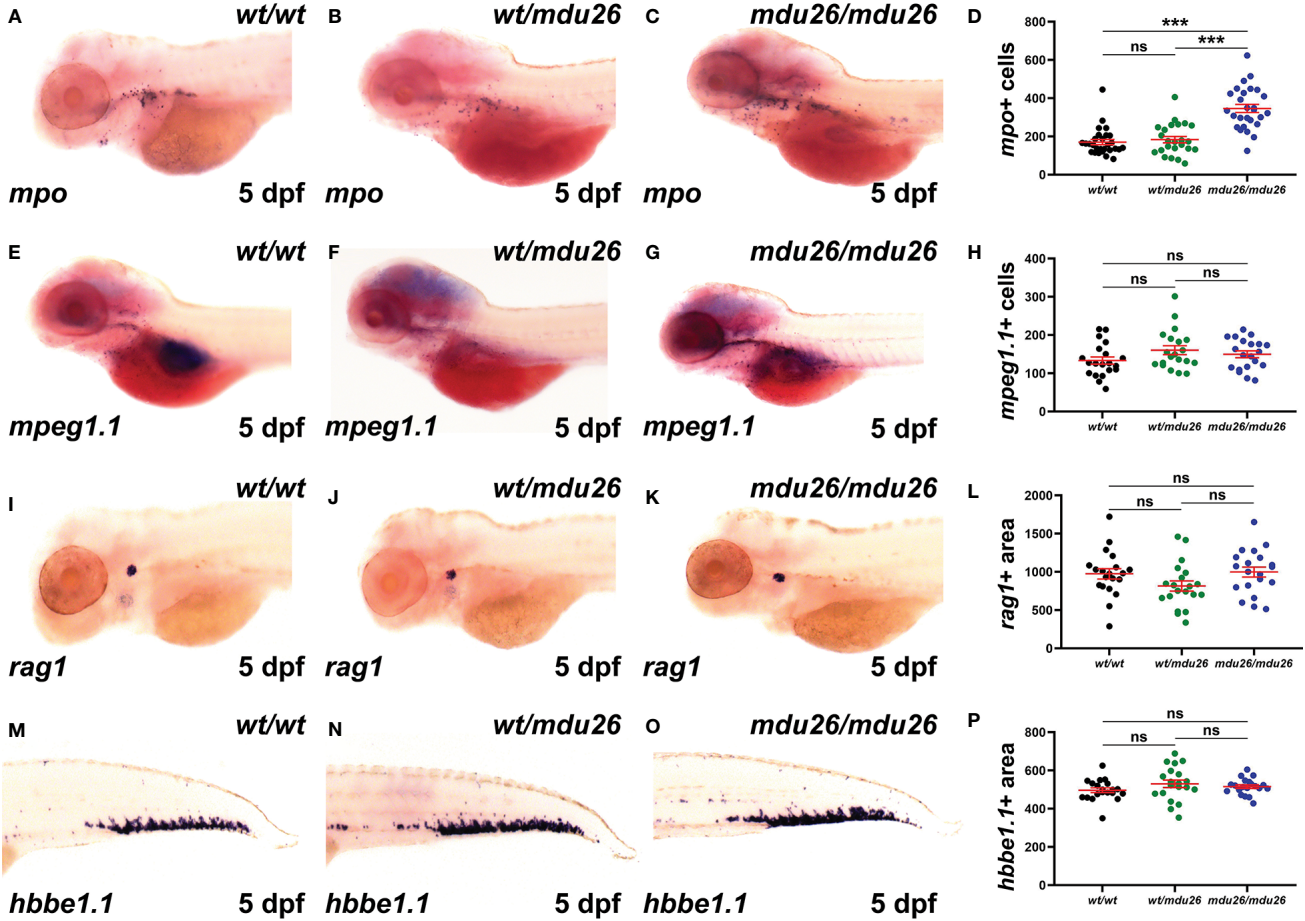
Effect of G-CSFR truncation mutation on definitive hematopoiesis. Wild-type (*wt/wt*), heterozygous (*wt/mdu26*) and homozygous (*mdu26/mdu26*) mutant *csf3r* embryos were subjected to WISH at 5 dpf with *mpo* **(A–C)**, *mpeg1.1* **(E–G)**, *rag1* **(I–K)** and *hbbe1.1* **(M–O)**, with representative images shown. Individual embryos were assessed for the number of *mpo*+ **(D)** or *mpeg1.1*+ **(H)** cells or the area of staining for *rag1* **(L)** or *hbbe1.1* **(P)**, with mean and SEM shown in red and level of statistical significance indicated (***: *p* < 0.001, ns: not significant; n=20-28).

Definitive hematopoiesis commences during the second day post-fertilization (dpf) and fully supplants primitive hematopoiesis by 5 dpf (22). At this time point, a significant increase in *mpo^+^* cells was observed in homozygous *csf3r^mdu26/mdu26^* compared to *csf3r^wt/wt^* embryos, but there was no longer a difference between heterozygous *csf3r^wt/mdu26^* and *csf3r^wt/wt^* embryos (**Figures 3A–D**). A similar elevation in *mpo* ^+^ cells was seen in homozygous *csf3r^mdu27/mdu27^* compared to *csf3r^wt/wt^* embryos (**Supplementary Figures 1D–F**). In contrast, there were no differences in the number of cells positive for *mpeg1.1*, a marker of macrophages (41) (**Figures 3E–H**), or the area of staining for *rag1*, a marker of T cells (42) (**Figures 3I–L**), or of *hbbe1.1*, a marker of mature erythrocytes (43) (**Figures 3M–P**).

### 3.4 Impact of G-CSFR truncation mutation on adult steady-state hematopoiesis

**Figure 4:**
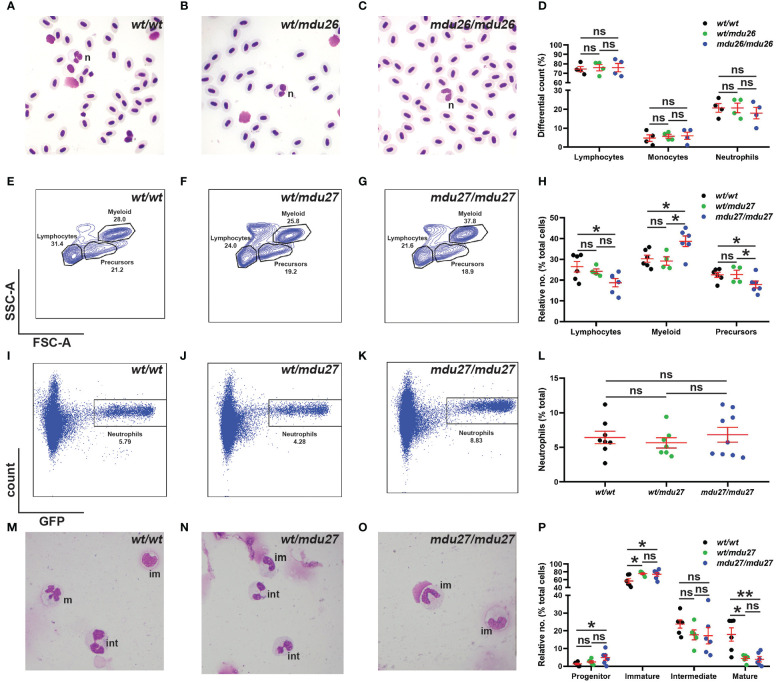
Effect of G-CSFR truncation mutation on adult hematopoiesis. **(A–D)**. Adult blood cells from wild-type (*wt/wt*), heterozygous (*wt/mdu26*) and homozygous (*mdu26/mdu26*) mutant *csf3r* fish were subjected to Giemsa-staining **(A–C)**, along with differential quantitation of the indicated blood cell populations for individual fish **(D)**, with mean and SEM shown in red and level of statistical significance indicated (ns: not significant; n=4). Abbreviation: n: neutrophil. **(E–P)**. Adult kidney cells from wild-type (*wt/wt*), heterozygous (*wt/mdu27*) and homozygous (*mdu27/mdu27*) mutant *csf3r* fish on a Tg(*mpo::GFP*) background were subjected to FACS analysis using SSC/FSC **(E–G)** and GFP fluorescence **(I–K)**, along with quantitation of indicated cell populations **(H)** and GFP+ neutrophils **(L)**, with sorted GFP+ cells subjected to Giemsa-staining **(M–O)**, along with differential quantitation of relative differentiation **(P)**. Panels **(H, L, P)** display results for individual fish, with mean and SEM shown in red and level of statistical significance indicated (*: *p* < 0.05, ns: not significant; n=4-9). im, immature; int; intermediate; m, mature.

Adult blood samples were subjected to histological analysis, which was quantified using differential counting. No significant differences were observed between *csf3r^mdu26/mdu26^*, *csf3r^wt/mdu26^* and *csf3r^wt/wt^* fish with regard to specific blood populations (**Figures 4A–D**), which was confirmed in *csf3r^mdu27/mdu27^* and *csf3r^wt/mdu27^* adults (**Supplementary Figures 1G–J**). The adult kidney marrow was further analyzed in *csf3r^mdu27/mdu27^* fish on the Tg(*mpo::GFP*) background using FACS. The SSC/FSC plot revealed a significant increase in the relative number of myeloid cells in, with other populations concomitantly decreased (**Figures 4E–H**). However, specific analysis of the GFP+ neutrophil population revealed no change in overall neutrophil numbers (**Figures 4I–L**). Moreover, histological analysis of sorted GFP+ cells revealed a decrease in relative maturity of neutrophils in both *csf3r^wt/mdu27^* and *csf3r^mdu27/mdu27^* compared to *csf3r^wt/wt^* fish (**Figures 4M–P**).

Since G-CSFR signaling has been implicated in cell survival (2), it was of interest to assess if this might explain the altered granulopoiesis. However, no difference was observed for early (AnnexinV+/7AAD-) and late apoptotic (AnnexinV+/7AAD+) populations between *csf3r^mdu27/mdu27^* and *csf3r^wt/wt^* fish in either the total myeloid (**Supplementary Figures 2A–D**) or GFP+ neutrophil (**Supplementary Figures 2E–H**) populations.

### 3.5 Impact of truncating G-CSFR mutants on emergency granulopoiesis

**Figure 5:**
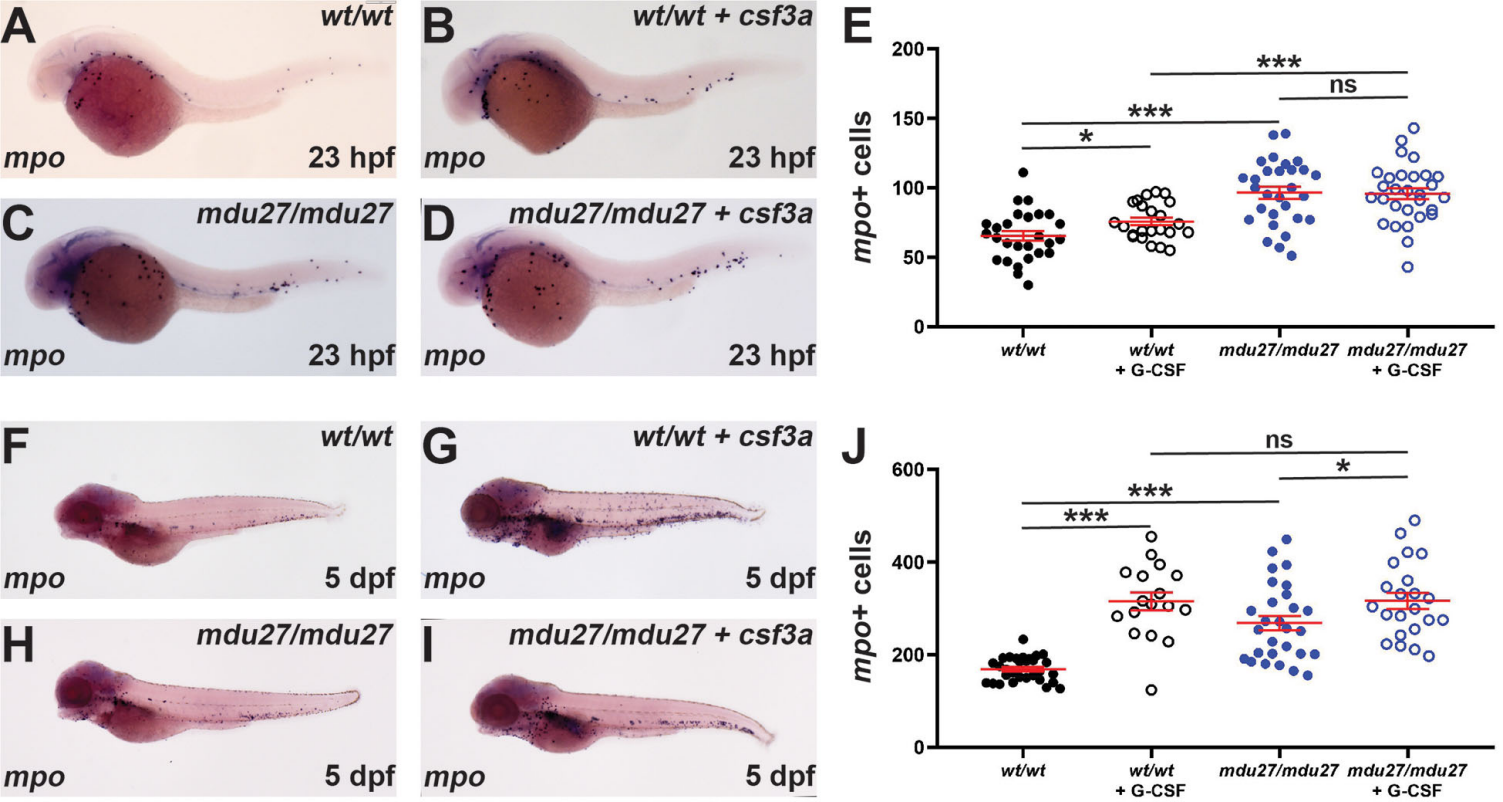
Effect of G-CSFR truncation mutation on emergency hematopoiesis. Wild-type (*wt/wt*) and homozygous (*mdu27/mdu27*) mutant *csf3r* embryos, either uninjected or injected with mRNA encoding G-CSF (+ *csf3a*) were subjected to WISH with *mpo* during primitive **(A–D)** and early definitive **(F–I)** hematopoiesis, with representative images shown. Individual embryos were assessed for the number of *mpo*+ cells for primitive **(E)** and definitive **(J)** hematopoiesis, with mean and SEM shown in red and level of statistical significance indicated (***: *p* < 0.001, *: *p* < 0.05, ns: not significant; n=17-30).

G-CSF is known for its key role in ‘emergency’ neutrophil production such as during an infection (1, 2). Moreover, G-CSF is directly administered to SCN patients to alleviate neutropenia (44). Therefore, it was important to identify the effects of G-CSFR intracellular truncation mutations on emergency hematopoiesis stimulated by G-CSF. Both *csf3r^mdu27/mdu27^* and *csf3r^wt/wt^* embryos were injected with *csf3a* mRNA encoding a zebrafish G-CSF or left uninjected, as previously described (3). WISH analysis revealed a significant increase in *mpo+* cells at 23 hpf following enforced G-CSF expression in *csf3r^wt/wt^* embryos (**Figures 5A, B, E**), as expected (3). Both uninjected and injected *csf3r^mdu27/mdu27^* mutants had significantly more neutrophils than the corresponding *csf3r^wt/wt^* embryos (**Figures 5A–E**), but there was no significant difference in neutrophil numbers in injected versus uninjected *csf3r^mdu27/mdu27^* embryos (**Figures 5C–E**). A significant increase in *mpo^+^* cells in injected compared to uninjected *csf3r^wt/wt^* embryos at 5 dpf was again evident (**Figures 5F, G, J**) and between uninjected *csf3r^mdu27/mdu27^* and both uninjected *csf3r^wt/wt^* (**Figures 5F, H, J**) and injected *csf3r^mdu27/mdu27^* embryos (**Figures 5H–J**), but not between the injected *csf3r^mdu27/mdu27^* and injected *csf3r^wt/wt^* embryos (**Figures 5G, I, J**). This was confirmed in *csf3r^mdu27/mdu27^* and *csf3r^wt/wt^* embryos on the Tg(*mpo::GFP*) background (**Supplementary Figure 3**).

## 4 Discussion

The G-CSFR has a pivotal function in the regulation of neutrophil production and function, as well as hematopoietic stem cell mobilization, particularly in ‘emergency’ situations (1, 2). Mutations that render the G-CSFR non-functional have been shown to result in profound neutropenia (45–47), which has been verified in mice (19, 48). In contrast, mutations that enhance G-CSFR signaling are associated with leukemias and other myeloproliferative disorders (11, 12). These include acquired truncation mutations first identified in SCN patients with a strong predisposition to the development of AML (5, 49), but also described in *de novo* and recurrent AML, as well as CNL, aCML and CMML patients (8–14). These mutations serve to truncate the G-CSFR intracellular domain, resulting in hyperresponsiveness to G-CSF (5, 18, 49). Previous studies have shown zebrafish possess a conserved G-CSFR, including key residues critical for intracellular signaling (3), with inactivating mutations of zebrafish G-CSFR able to reproduce the sustained neutrophil deficiency observed in SCN (25, 26). This study aimed to create truncating G-CSFR mutations in zebrafish using CRISPR-Cas9-mediated genome editing and to characterize their impact on primitive, definitive and emergency hematopoiesis.

Three mouse models of truncated ‘hyperresponsive’ G-CSFR mutants have been generated. One in which the endogenous mouse gene was mutated (19) and another in which a truncated human G-CSFR was expressed transgenically (21) showed decreased peripheral neutrophils, although the bone marrow contained normal neutrophil numbers and an elevated number of immature myeloid cells. In contrast, an alternate mouse model with targeted mutation of the endogenous gene showed normal circulating neutrophil numbers, but elevation of band and segmented neutrophils as well as progenitors in the bone marrow (20). The results in zebrafish adults are consistent with this last mouse model, with the number of peripheral neutrophils not significantly different between genotypes, but those in the kidney marrow exhibiting reduced maturation. This zebrafish study has allowed for the first time to assess the effect of hyperresponsive truncating G-CSFR mutations on primitive and early definitive hematopoiesis. Strikingly, during primitive hematopoiesis, neutrophil numbers were elevated. While this is clearly different to what is observed in adult mice and zebrafish, it should be noted that G-CSF/G-CSFR signaling in zebrafish has been shown to be particularly important for primitive neutrophil production, with ablation of either leading to an almost complete loss of this population (3, 26). This developmental stage is therefore perhaps more comparable to the situation when adult mice are injected with G-CSF, when significant increases in neutrophils are observed. In contrast, during early definitive zebrafish hematopoiesis the effects of G-CSFR truncation were less marked, consistent with previous studies showing the G-CSFR ablation has a more modest impact on zebrafish neutrophils during this phase (3, 26). All mouse models noted hyperresponsive to exogenous G-CSF resulting in increased production of cells along the neutrophil lineage (19–21). This collectively illustrates the complex regulation of neutrophil production mediated by G-CSFR.

Truncated G-CSFR mutants have been described extensively as exerting a ‘dominant-negative’ effect on wild-type receptors (5). However, the data presented here indicated that the truncating *csf3r* allele was not strictly dominant over the wild-type allele. Notably, heterozygous mutants were similar to homozygous mutants with respect to primitive hematopoiesis, but more like wild-type fish with respect to early definitive hematopoiesis and variable in adults. Moreover, close examination reveals that this is also the case in other studies. For example, while heterozygous and homozygous mutant mice showed equivalent initial responses to G-CSF injection, these were more sustained in homozygote animals (50). In other studies, cells expressing both normal and truncated G-CSFR exhibited intermediate phenotypes or responses, including with respect to sustained signaling and impaired internalization (17, 50). Thus, truncating G-CSFR mutations should be more accurately described as ‘co-dominant’. Indeed, there are good biochemical rationale as to why this may be. Firstly, since functional receptor complexes consist of multimers, a relevant cell in a heterozygous individual will express a mixture of receptor forms on their surface, a proportion of which will contain only wild-type receptors able to signal normally. Secondly, those receptor complexes that contain at least one wild-type receptor chain will still interact with the normal regulatory machinery. Thirdly, the enhanced signaling in heterozygote cells is likely associated with increased expression of SOCS3, a key negative regulator of the G-CSFR (51), enhancing its ability to act on those receptor complexes carrying a wild-type G-CSFR chain, since it retains the requisite SOCS3 binding site (52).

Zebrafish expressing truncated G-CSFRs did not exhibit overt leukemia, which is also true of mouse models (19–21), confirming that co-operating genes are absolutely required. In patients who have acquired AML subsequent to SCN, a number of common genetic lesions have been identified, including partial or complete loss of chromosome 7 and activating *RAS* mutations (53, 54). Inactivating mutations in *RUNX1* (55, 56) and *CEBPA* (57) as well as the *PML-RARa* oncoprotein fusion (58) have been reported to cooperate with truncated hyperresponsive G-CSFR mutations. In addition, STAT5, which shows particularly strong sustained activation by truncated G-CSFRs (17), has been implicated in mediating the effects of truncated G-CSFRs (59). The zebrafish model that was generated in this study will be highly valuable in analyzing additional co-operating genes.

A range of other G-CSFR mutations have been identified that are associated a variety of leukemias and myeloproliferative disorders (5). These include activating mutations such as T618I in CNL and related disorders (11, 55, 60), and a E785K polymorphism associated with MDS (61). These exert distinctive pathological effects, activate different (and sometimes overlapping) pathways (11, 60), and have been demonstrated to respond differentially to pharmacological agents. For example, the JAK2 inhibitor ruxolitinib has been shown to decrease proliferative signaling and neutrophil count in patients with G-CSFR-T6181 mutations whereas the SRC inhibitor dasatinib (but not ruxolitinib) was able to selectively inhibit proliferation of cells expressing truncated G-CSFRs (11). Analyzing the array of pathogenic G-CSFR mutants in zebrafish provides an opportunity to explore the specificity of their action, downstream pathways and therapeutic sensitivity in the context of a whole animal, which is an exciting prospect. Finally, the truncating mutations often occur concurrently with these (and other) G-CSFR mutations (11, 55, 62), which could also be investigated in zebrafish.

This study has successfully created and characterized a zebrafish model of hyperresponsive G-CSFR truncations. Significantly enhanced neutrophil production was observed throughout successive waves of embryonic hematopoiesis, but a defect in neutrophil maturation was also evident in adult kidney marrow. The G-CSFR truncation also displayed partial dominance. Collectively, this has shed new light on the impact of such mutations across the lifespan and generated a *bone fide* zebrafish model to facilitate further clinically-relevant studies to explore the role of co-operating mutations and genes, as well as downstream effector pathways that may inform new approaches to therapy, which could be readily trialed in this model.

## Data availability statement

The original contributions presented in the study are included in the article/**Supplementary Material**. Further inquiries can be directed to the corresponding author.

## Ethics statement

The animal study was reviewed and approved by Deakin University Animal Ethics Committee.

## Author contributions

AW conceived the project. FB and VB generated the zebrafish G-CSFR mutants and VB and MG performed most of the *in vivo* and *in vitro* characterization. AW and CL co-supervised VB, MG and FB and contributed to on-going experimental design. All authors contributed to the article and approved the submitted version.
