# Supplementary material for: Leukemia-associated truncation of granulocyte colony-stimulating factor receptor impacts granulopoiesis throughout the life-course
Source: Front Immunol. 2023 Jan 10;13:1095453. doi: 10.3389/fimmu.2022.1095453 (PMC9871641; doi:10.3389/fimmu.2022.1095453)

Supplementary Figure 1. Effect of *mdu27* allele on primitive and definitive hematopoiesis. Wild-type (*wt/wt*) and homozygous (*mdu27/mdu27*) mutant *csf3r* embryos were subjected to WISH with *mpo* at 23 hpf (A-B) and 5 dpf (D-E). Individual embryos were assessed for the number of mpo^+ at 23 hpf (C) and 5 dpf (F), with mean and SEM shown in red and level of statistical significance indicated (***: $p < 0.001$; n=28-32). Adult blood cells from wild-type (*wt/wt*), heterozygous (*wt/mdu27*) and homozygous (*mdu27/mdu27*) mutant *csf3r* fish were subjected to Giemsa-staining (G-I), along with differential quantitation of the indicated blood cell populations for individual fish (J), with mean and SEM shown in red and level of statistical significance indicated (ns: not significant; n=6). Abbreviation: n: neutrophil.

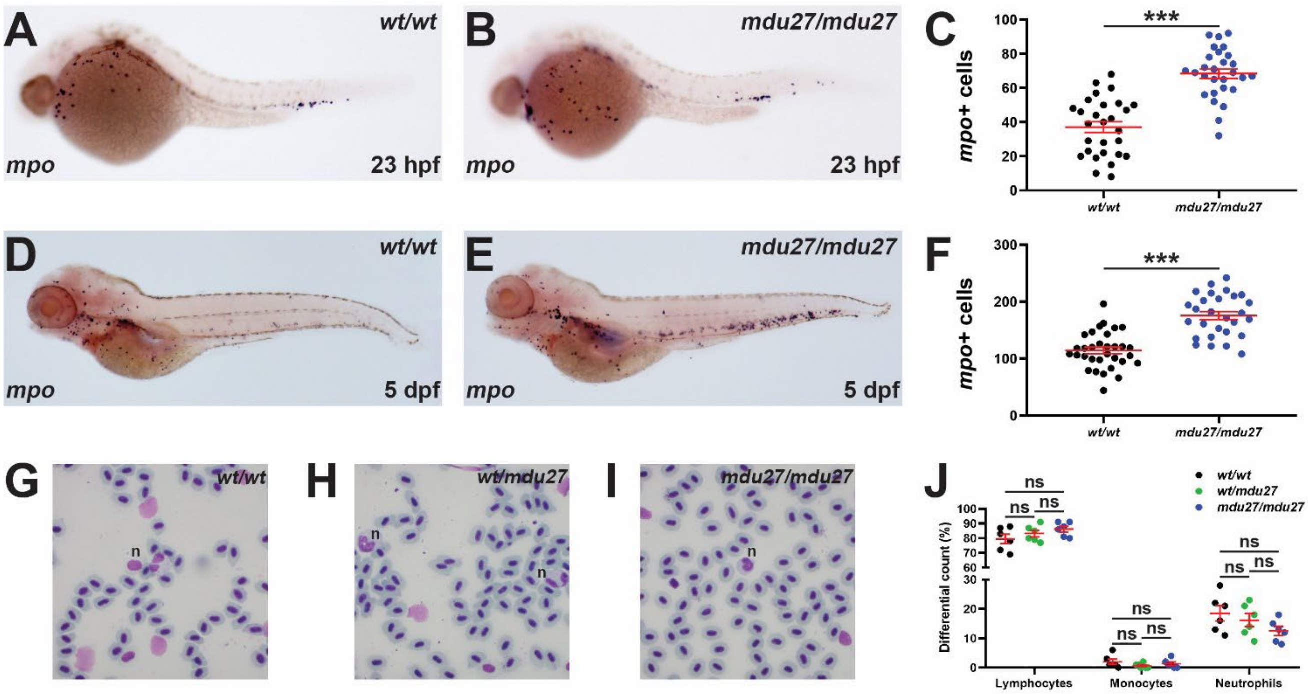

Supplementary Figure 2. Effect of truncating G-CSFR mutation on apoptosis in adult kidney marrow. Kidney cells from wild-type (*wt/wt*), heterozygous (*wt/mdu27*) and homozygous (*mdu27/mdu27*) mutant *csf3r* adult fish were subjected to Annexin V/7-AAD staining on gated myeloid cell population (A-C) or GFP+ neutrophil population (E-G), with representative samples shown. Individual fish were quantified with respect to specific quadrants in the myeloid (D) and GFP+ neutrophil (H) analyses, with mean and SEM shown in red and level of statistical difference indicated (ns: not significant; n=4-6).

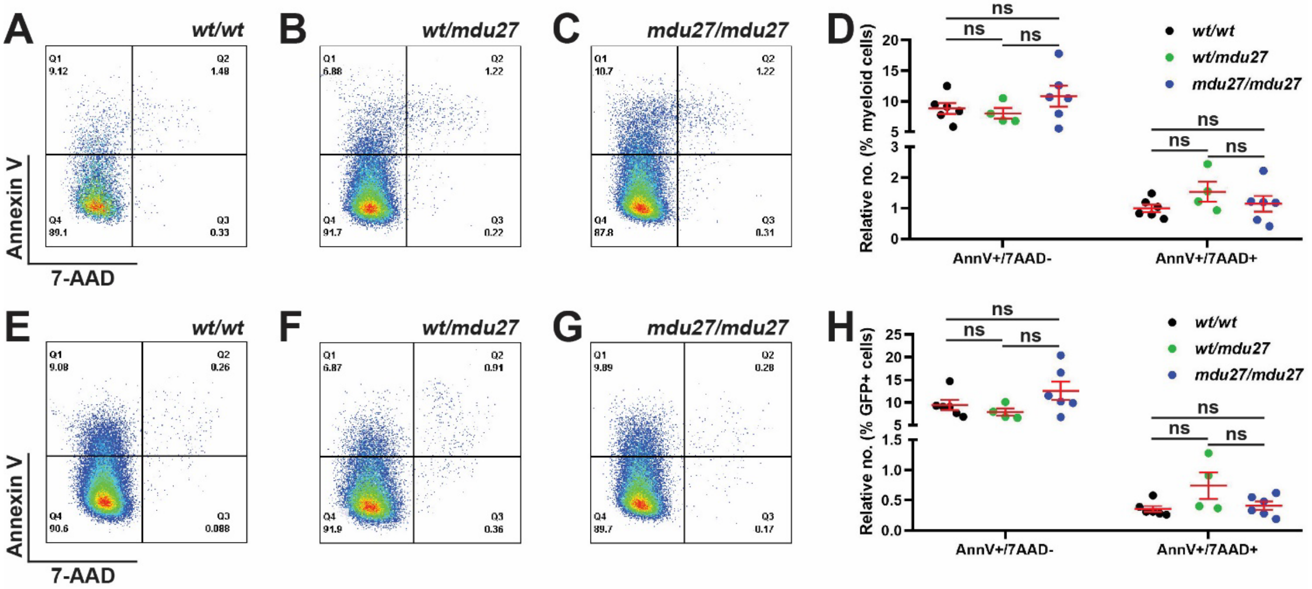

Supplementary Figure 3. Effect of G-CSFR truncation mutation on emergency hematopoiesis in transgenic zebrafish. Wild-type (*wt/wt*) and homozygous (*mdu27/mdu27*) mutant *csf3r* embryos on the Tg(*mpo::GFP*) background, either uninjected or injected with mRNA encoding G-CSF (+ *csf3a*) were fluorescently imaged during early definitive hematopoiesis with representative images shown (A-D). Individual embryos were assessed for the number of *mpo*+ cells (E), with mean and SEM shown in red and level of statistical significance indicated (***: $p < 0.001$, *: $p < 0.05$, ns: not significant; n=24-29).

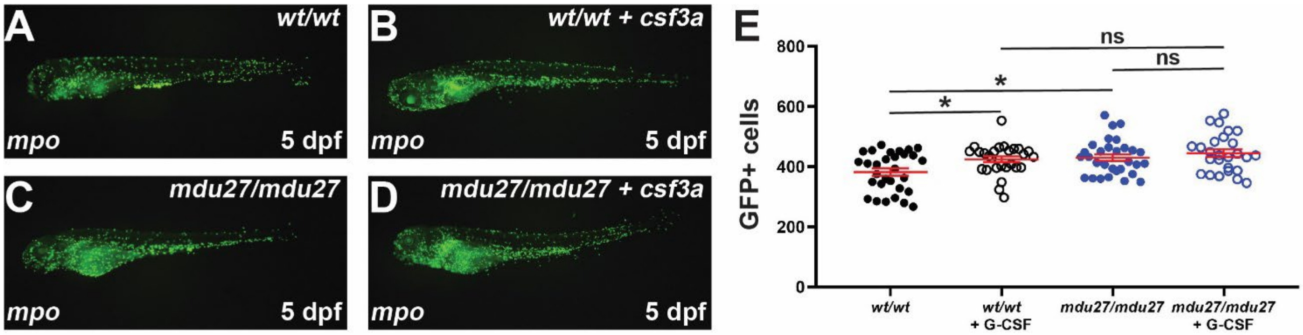

Supplement: Supplementary file 1 [file DataSheet_1.pdf]
